# Parental experiences and coping strategies when caring for a child receiving paediatric palliative care: a qualitative study

**DOI:** 10.1007/s00431-019-03393-w

**Published:** 2019-05-19

**Authors:** Lisa M. Verberne, Marijke C. Kars, Antoinette Y. N. Schouten-van Meeteren, Esther M. M. van den Bergh, Diederik K. Bosman, Derk A. Colenbrander, Martha A. Grootenhuis, Johannes J. M. van Delden

**Affiliations:** 1 Department of Pediatrics, Maastricht Universtity Medical Centre, P. Debyelaan 25, 6229 HX Maastricht, The Netherlands; 2 Department of Medical Humanities, Julius Center for Health Sciences and Primary Care, University Medical Center Utrecht, Heidelberglaan 100, 3508 GA Utrecht, The Netherlands; 3 Princess Máxima Center for Pediatric Oncology, Lundlaan 6, 3584 AE Utrecht, The Netherlands; 4 Department of Pediatrics, Emma Children’s Hospital, Amsterdam University Medical Center, Meibergdreef 9, 1105 AZ Amsterdam, The Netherlands

**Keywords:** Paediatrics, Palliative care, Parents, Caregiving, Experiences, Coping

## Abstract

Parenting and providing extensive care to a child with a life-limiting or life-threatening disease while being aware of the future loss of the child are among the most stressful parental experiences. Due to technical and medical improvements, children are living longer and are increasingly cared for at home. To align healthcare professionals’ support with the needs of parents, a clear understanding of prominent experiences and main coping strategies of parents caring for a child in need of palliative care is needed. An interpretative qualitative study using thematic analysis was performed. Single or repeated interviews were undertaken with 42 parents of 24 children with malignant or non-malignant diseases receiving palliative care. Prominent reported parental experiences were daily anxiety of child loss, confrontation with loss and related grief, ambiguity towards uncertainty, preservation of a meaningful relationship with their child, tension regarding end-of-life decisions and engagement with professionals. Four closely related coping strategies were identified: suppressing emotions by keeping the loss of their child at bay, seeking support, taking control to arrange optimal childcare and adapting to and accepting the ongoing change(s).

*Conclusion*: Parents need healthcare professionals who understand and carefully handle their worries, losses, parent-child relationship and coping strategies.

**Table:** 

**What is Known:** • *In paediatric palliative care, parents have a daunting task in fulfilling all caregiving tasks while striving for control of their child’s symptoms, a life worth living and a family balance.*	
**What is New:** • *Prominent experiences were: continuous management of anxiety of child loss, feelings of uncertainty, tension with end-of-life decision making and engagement with professionals. Parents experienced unique significance to their child, reinforcing a meaningful parent-child relationship.* • *Relevant coping strategies were: suppressing emotions, seeking support, taking control to arrange optimal care and adapting to the ongoing changes.* • *To provide tailored support, professionals need to understand parents’ perceptions, relationship with their child and coping strategies.*

**Electronic supplementary material:**

The online version of this article (10.1007/s00431-019-03393-w) contains supplementary material, which is available to authorized users.

## Introduction

Children with life-limiting or life-threatening diseases (LLD/LTD; Box 1) are increasingly cared for at home. At the same time, the period in which paediatric palliative care (PPC) needs to be provided by the parents is becoming increasingly longer due to technical and medical improvements [9, 17, 25, 32, 47]. The caregiving role of the parents is extensive and complex [13, 14, 20, 48, 54]. In addition to routine childcare, caregiving also includes a range of demanding emotional, technical and nursing tasks [13, 20, 42, 48, 53, 54].

Box 1 Definitions [1]

**Table:** 

Life-limiting disease: conditions for which are that there is no reasonable hope of a cure and from which children or young people will die. Life-threatening disease: conditions for which are that curative treatment can be feasible but can fail.	

Studies indicate that parents of a child with a LLD/LTD are often exhausted, suffer from emotional and physical distress and report below average quality of life [4, 35, 40, 54]. They also have an increased risk of post-traumatic stress disorder [10, 18, 33, 49]. Many parents know that at some point in the future, they will lose their child, which means they have to manage the extraordinary situation of providing care to a child living under the ‘shadow of death’. As such, parents face the enormous challenge of being a parent and providing the best care to their child, while at the same time processing their situation and acting accordingly [46].

A better understanding of parents’ experiences when caring for a child with a LLD/LTD at home, and how they adjust to their situation, is needed. It is hypothesised that this knowledge will improve HCPs’ ability to align their support with the parents’ perspectives and needs. In the PPC literature, most articles are based on paediatric cancer patients; however, 60% of the children with a LLD/LTD have non-malignant diseases [1, 11, 15, 26]. Therefore, this study aims to provide insight into the most prominent experiences of parents caring for a child with a malignant or non-malignant LLD/LTD at home and to identify the main coping strategies they adopt to allow themselves to continue with their daily lives.

## Methods

An interpretive qualitative study using an inductive thematic analysis was conducted to examine experiences and coping strategies from the perspective of parents caring for a child receiving PPC [3, 5, 43]. The method and reporting follow the Consolidated Criteria for Reporting Qualitative Research (COREQ) [45].

### Sample

A purposive sample was recruited comprising Dutch-speaking parents (age ≥ 18 years) of children with a LLD/LTD, primarily residing at home, who were referred to the Emma Children’s University Hospital paediatric palliative care team (PPCT). Referral to the PPCT ensured a general agreement among HCPs that such palliative care was indicated. PPCT referrals thus provided access to families of children with a variety of diseases. These families were in the best position to maximally inform us about our focus of interest: parenting and caregiving in PPC [27]. To capture a wide range of perspectives, variation in the children selected was sought with respect to age (0–18 years), malignant (MD) and non-malignant (NMD) diagnoses and the phase of the palliative trajectory. Four phases of the palliative trajectory were distinguished in the literature: diagnosis, loss of normality and adjusting to a new normality, declining and the dying phase [14, 52]. Some parents were approached soon after the child’s death to gain deeper insight into parenting and caregiving during the end-of-life and dying phase. Parents of 35 children were identified as eligible. Either the physician treating the child or a PPCT member introduced this study to parents and asked permission for the researcher to contact them. In six eligible cases, the HCP introducing the study assessed the parents’ situation as too vulnerable to inform them about this study. Parents of 29 children were contacted by telephone and invited to participate. In five cases, parents refused participation. The reasons for refusal were as follows: no time (*n* = 2), too burdensome (*n* = 2) or unknown (*n* = 1).

### Data collection

Individual semi-structured, in-depth interviews were conducted at home. The interviews lasted between 30 min and 2 h and were conducted between August 2013 and November 2015. The interviewers (LV, MB: trained in qualitative research; MK: experienced qualitative researcher) were independent researchers from a university hospital other than that from which the parents were recruited and were, therefore, not involved in the treatment of the child or known to the family. To check whether all issues were discussed, a topic list (Online Supplement) based on the literature and expert knowledge was used during each interview. Topics relevant for this study included parenting, caregiving and goal setting, professional care, coping and family life. Field notes were made after each interview. Interviews were audio recorded and transcribed verbatim. The study was approved by the research ethics committee of the Academic Medical Centre Amsterdam (June 12, 2013; reference number W13_120 # 13.17.0153). Written informed consent was obtained from all the parents participating.

### Data analysis

An inductive thematic analysis was used in accordance with methods that optimise validity and rigour [3, 5, 28, 43]. Three researchers (LV, MK and JvD) were involved throughout the entire process. They used joint meetings to reach agreement on interpretation of the data and findings and worked towards consensus. Therefore, researcher triangulation was ensured to improve the reliability and validity of the analysis.

The thematic analysis consisted of two phases. Firstly, the researchers (re)read the transcripts of eight interviews to become familiar with the content of the interviews [3, 5, 43]. At least two researchers individually analysed and coded these eight transcripts with paper and pencil and compared interpretations together. The meaning of the separate fragments was determined by interpreting them in light of the complete transcript [5, 22]. The initial codes were recoded resulting in an adapted code list with themes and concepts on a more abstract and conceptual level [5].

Secondly, every new interview was read and discussed by at least two researchers. One researcher (LV) and a trained research assistant coded all transcripts, supported by the software program NVivo10 [31]. After coding each transcript, the code tree was evaluated and, if indicated, revised. The separate codes were sorted into potential themes, which were defined and refined [3].

To guide the constant comparative method, the research team went back and forth between the different steps and the entire data set to capture the key aspects of the themes in the raw transcripts [5]. An audit trail was used to record methodological choices and substantive ideas and concepts related to the interpretation of the data. This enabled the validity of the results to be ensured and provided their transparency. Saturation was reached on a conceptual level. External validation of the results was provided by two parent association representatives, respectively, for children with malignant and non-malignant diseases. In addition, the results were checked in an expert meeting among nine HCPs experienced in paediatrics, PPC and/or homecare. This led to only minor adjustments of the results.

## Results

In total, we included 24 mothers and 18 fathers of nine children with a MD and 15 children diagnosed with a NMD. A total of 47 single or repeated interviews were conducted. Eleven parents were interviewed after their child’s death, of which five interviews were second interviews. For patient characteristics, see Table 1. Representative quotations were chosen to illustrate the experiences and coping strategies identified (Tables 2 and 3). Despite differences in disease trajectories, the experiences and coping strategies described below were quite similar for parents of both the MD and NMD groups.

**Table 1:** Characteristics of parents (*n* = 42) and their ill child (*n* = 24)

Characteristics	Number (*N*)	Percentages (%)
Gender parent		
Male	18	43
Female	24	57
Age parent^a^		
< 30	2	5
30–40	29	73
> 40	9	23
Marital stage		
Married/cohabiting	38	90
Divorced/not cohabiting	4	10
Education		
Low^b^	5	12
Middle^c^	15	36
High^d^	22	52
Age child at first interview (years)		
0–1	1^e^	4^e^
1–5	13^f^	54^f^
5–12	7	29
12–16	2	8
≥ 16	1	4
Child gender		
Male	12	50
Female	12	50
Child diagnosis		
Non-malignant disease (total)	15	63
Congenital anomalies	11	46
Neurodegenerative disease	2	8
Metabolic disease	2	8
Malignant disease (total)	9	38
Central nervous system tumour	5	21
Bone/soft tissue sarcoma	2	8
Neuroblastoma	1	4
Leukaemia	1	4
Time since diagnosis		
0–6 months	2	8
6–12 months	3	13
1–2 years	7	29
2–5 years	8	33
> 5 years	4	17
Palliative phase at first interview		
Diagnostic phase	0	0
Phase of loss of normality	15	63
Phase of decline	6	25
Dying phase	3	13
Siblings per case		
0	5	21
1	11	46
2	7	29
3	1	4

Percentages may not equal 100 due to rounding

^a^Age of two parents is missing

^b^Low: primary school, lower secondary general education, lower vocational education

^c^Middle: higher secondary general education, intermediate vocational education

^d^High: higher vocational education, university

^e^On one case, the interview took place after the child’s death

^f^In two cases, the interview took place after the child’s death

**Table 2:** Quotes that illustrate parental main experiences in caring for their child with a life-limiting or life-threatening disease at home

	(Sub)Theme	Quote
Daily anxiety of child loss		
1A	Daily anxiety	Case 4: boy, 10 years old, MD. Mother: *You are, of course, busy with it every day. There are moments, every day, when you wonder if in the furure, Mees (son) will still be here then? And the better he is, you become more afraid of receiving bad news. Yes, you can suffer great setbacks. By now we know we will have to endure further serious setbacks again. That makes me… That thought is with me every day.*
1B	New symptoms	Case 7: boy, 9 years old, NMD. Mother: *As soon as he stops coughing and his temperature rises, yes then you do not get nervous in the sense that I panic, but you surely hold your breath for… each time… Is it now? Will this be the moment that….?*
1C	Taking no risks	Case 8: boy, 6 years old, NMD. Father about life-threatening situations: *That is why you are so careful with him, to prevent a respiratory tract infection, because you have seen what can happen, or worse. When the sun is shining and it is 15 degrees and you walk with a rain cover on the buggy, then people can often stared at as if you are crazy. While I’m thinking that I just want to do everything to try to prevent that moment.*
Confrontation with loss and related grief		
2A	Deterioration of the child	Case 7: boy, 9 years old, NMD. Father: *It is very sad to literally see your child declining, while you cannot really do anything. It’s so difficult to see your child slip away before your eyes…*
2B	Losing family life	Case 4: boy, 10 years old, MD. Mother: *And, meanwhile seeing Freek (sibling) playing in the water and splashing around and you are so used to both of them (the sibling and ill child) doing it all together. Yes, it is really, really tough to see that Mees cannot actually do anything anymore. And so Freek has to start playing on his own. That will soon be reality. You can see this all the time.*
2C	Loss of carefree family life	Case 14: girl, 1 year old, NMD. Father: *She continues to lag behind in motor skills and development. A boy born three weeks later than Maaike (daughter) lives across the street. I’m deeply saddened to compare her with him. He recognises you, he waves at you, he already walks. These things are very painful.*
2D	Losing the future you dreamed of	Case 17: girl, 9 years old, MD. Mother: *And of course you always had prospects of the future but, actually, since the moment that she became sick, that was – bang – all at once taken away.*
Ambiguity towards uncertainty		
3A	Uncertain prognosis	Case 8: boy, 6 years old, NMD. Father: *Neither were the doctors able to say anything about that (son’s life expectancy) because there are so few children with this disease. Even when the doctors relatively know a lot, they still only have around five children under their treatment. […] I really found that difficult in the beginning.*
3B	Living with uncertainty	Case 6: boy, 2 years old, NMD. Father: *A parent should not outlive their child. And that is happening right now. It can also happen to my other children, but you know that this will happen. It may be this year, it may be next year but really, no longer. This, therefore, occupies my thoughts.*
3C	Ambiguity	Case 13: boy, 5 years old, MD. Mother: *That uncertainty, the fact that you know it, but that it does not happen, is an enormous burden I have to carry with me. Sometimes you almost wish it would happen, then you would have it behind you. But then, Max (son) would no longer here… So that is not possible either, it just cannot happen.*
Preservation of a meaningful parent-child relationship		
4A	‘Being there’	Case 19: boy, interview took place after the child’s death, MD. Mother: *If things go really bad for your child, then you just do not want to perform the care for your child any more, you just want to be there. […] Just, purely and simply really being there for your son, cuddling him, hugging him.*
4B	Being a ‘good parent’	Case 6: boy, 2 years old, NMD. Father: *If he (son) dies, I want to be able to look in the mirror and say: “Bob, you have done your best for him”, just as I would for a healthy child. I do not have a healthy child but I want to feel that I can respect myself for having done my best. It is my child. It is my responsibility.*
Tension regarding end-of-life decisions		
5A	Comfort	Case 6: boy, 2 years old, NMD. Father: *Once he (son) becomes ill, then it is simply a question of will he recover, or not. And if he does not recover, then you have to arrange other things. Then you need to let him die comfortably.*
5B	Looking ahead to the child’s last days	Case 14: girl, 1 year old, NMD. Mother: *I want the best for Maaike (daughter), but neither do I want to lose her. I can imagine that it will be very difficult when it gets near the end…(cries) You really want the best for Maaike and perhaps that best is not to carry on treating her endlessly. At the same time, that, of course, it is part of how I feel inside because I just do not want to lose her.*
5C	Child’s ‘choice’	Case 11: girl, interview took place after the child’s death, NMD. Father: *In our experience, she (daughter) has given up by herself at a moment. And that is surely more pleasant than to say OK… We now have the idea that she actually has done well.*
Engagement with healthcare professionals		
6A	Positive aspect	Case 5: boy, 15 years old, MD. Father: *She (homecare nurse) has done very well in my opinion. She has begun things and picked up on things, such as when we have to do things and how things are going with the contacts. So she helped push things along which I think is good - it feels good. Of course, they often deal with terminally ill children and children in this end-of-life phase, so you see too that bit of experience. And yes, every child is different, of course, but if I stand back and watch then you feel “this is OK”. It is a good feeling.*
6B	No treatment available	Case 7: boy, 9 years old, NMD. Mother: *Because in my son’s case, his diagnosis, it is just symptom management and that is all. […] And that is just so strange. Because you no longer have a medical route to follow or an idea that we are going to do something… you just go home.*
6C	Learning medical language of doctor	Case 13: boy, 5 years old, MD. Father: *I know our oncologist had already said that if it (cancer) comes back, things would look very bad. And, at a certain moment, you realise what that means. Because if it really looks bad, they actually mean that he (son) will not survive.*

Some quotes are slightly modified in order to improve readability. Names are fictitious

*MD* malignant disease, *NMD* non-malignant disease

**Table 3:** Quotes that illustrate the parental coping strategies when caring for their child with a life-limiting or life-threatening disease at home

	(Sub)Theme	Quote
Suppressing emotions		
1A	Keeping thoughts about child’s death out at bay	Case 13: boy, 5 years, MD. Father: *You have been through a lot with the boy, but yeah, death is obviously something, that you have kept out all this time, and it is irreversible… Yes, for me that is unknown territory… That scares me.*
1B	Enjoying here-and-now	Case 15: girl, 2 years, NMD. Mother: *Each day you can take it as it comes… one day at a time. And you can focus on it, but that really does not work because then your life stops. You must continue to enjoy things, to make a start on things. Then once she goes an extra day to day care. You see you have your own life too, and that is the way you should handle it. In truth, I just take one day at a time, thinking there is no time like the present.*
1C	Positive thinking	Case 19: boy, interview took place after the child’s death, MD. Father: *That you once again try to continue and try to stay positive, for Jelle (son) and, of course, for yourself in order to get through the process again.*
Seeking support		
2A	Peers	Case 7: boy, 9 years, NMD. Mother: *In one aspect what is good is that the Facebook community gives us a lot of support. When you are faced with: What could this be? Or: What kind of medication is needed?, then there is always someone who just has the same question, so other people can answer your questions. Because we, as parents, as a parent group…. with these diseases, we know a lot more than most doctors. So we can benefit from each other. But, then, yeah, the children who are facing death also come along. That is very confronting.*
2B	Distraction	Case 15: girl, 2 years, NMD. Mother: *Your world gets smaller, but when I work, I can really escape from it. You just have your work, your responsibilities at your work and that gives peace, that is relaxation. That sounds very strange, but work is now relaxation. A moment away, a moment with your mind in a different place. A moment with fewer worries to think about.*
2C	Religion/Faith	Case 5: boy, 15 years, MD. Father: *But we feel happily comforted, by the fact that we will see Pieter (son) again. That does not make the situation any less painful or sad, but it is a comfort in the long term. [..] How privileged I am to know that there is another way.*
Taking control		
3	Taking control	Case 4: boy, MD, interview took place after the child’s death. Father about the coordination and direction of care: *And they (homecare nurses) made it seem that it was all arranged in this way, but in practice, it was not crystal clear. Me and Anke (mother) felt we had to lose our temper in front of the general practitioner with the paediatric homecare organisation before they understood our boundaries, like, so you know, till here and no further. You (homecare nurses) are just going to do what the oncologist says and if you have doubts… then we will call the paediatric palliative care team now. And we did and, of course, it sounds very arrogant, but we were right. I said, well that is the first and last time that we do this.*
Adapting and accepting		
4A	Accepting	Case 9: girl, 3 years, NMD. Father: *So, yes, at a certain moment you just have to accept that life is sometimes like that.*
4B	Adapting one’s own life	Case 17: girl, 9 years, MD. Mother: *She no longer goes to after-school care because I feel something like ‘no, come home, I cannot miss you anymore’. I started to work less, to be at home with the children.*
4C	Feeling privileged	Case 8: boy, 6 years, NMD. Mother: about caring for her son*: I always say I that am lucky that he does not attend school. You hear quite often, my last (child) also attends school now; I do not have that.*
4D	Acceptance in relation to one’s own life	Case 7: boy, 9 years, NMD. Father*: Every time you have to cross a threshold yeah… a wheelchair is a threshold.. that little car was a whole complete drama, we had a wonderful sport utility vehicle, then you have to use that stupid wheelchair van. Every time you have to cross another threshold. And then you occasionally need a little push.*
4E	Accepting the course of the disease and the loss it brings	Case 14: girl, 1 year, NMD. Mother: *It is the same with walking, it is okay that she will not walk, I lift or push her. It does not matter. It does not make her any the less for it… I still love her just the same. But I did expect that maybe one day she would crawl from A to B herself, and now you are told that that will not happen either. Every time you are forced to cross that threshold, you have to accept it yourself. So it is a process in accepting another piece of loss.*

Some quotes are slightly modified in order to improve readability. Names are fictitious

*MD* malignant disease, *NMD* non-malignant disease

### Experiences

Six main experiences were identified from the interviews: (1) daily anxiety of child loss; (2) confrontations with loss and related grief; (3) ambiguity towards uncertainty; (4) preservation of a meaningful parent-child relationship; (5) tensions regarding end-of-life decisions; and (6) engagement with professionals.

1. *Daily anxiety of child loss*

At the moment, it became clear that their child was seriously ill, all parents felt confronted with their child’s vulnerability and questioned whether their child was going to die or not. Due to the life-limiting character of their child’s disease, parents felt forced to live with the harsh reality that sooner or later they will lose their child. Many parents mentioned that they were not supposed to outlive their children and so the acknowledgement that an early death was inevitable felt inhumane. All parents feared the moment of definite loss. Moreover, parents experienced thoughts about definitely losing their child came, more or less consciously, every day. This resulted in feelings of anxiety that existed irrespective of the expected life span or phase of the disease (Table 2, quote 1A). For many parents, in both MD and NMD group, these feelings of anxiety were increased by the fact that they had already faced the concrete threat of losing their child due to a severe, but intermittent, decline in the child’s physical condition or as a consequence of episodes of intensive treatment (e.g. chemotherapy, surgery). They realised that, at any moment, the progression of the disease, or a sudden crisis, could result in the definite loss of their child.

Several conditions deepened latent fear, such as overt information from HCPs indicating disease progression or entering the end-of-life phase. The introduction of a PPCT, as well as being confronted with end-of-life issues not directly related to their child, such as in newspapers or on television, was experienced as a confirmation with their harsh reality. Moreover, many parents experienced talking about the definite loss of their child as very distressing. All aforementioned conditions were experienced as extremely painful because it definitely confirmed that their child was seriously ill and/or about to die, which triggered immense anxiety.

Due to the anxiety of losing their child, parents became highly sensitive to any signal that could reflect the beginning of the end of their child’s life (Table 2, quote 1B). Therefore, most parents felt the urge to protect their child’s vulnerable condition as far as they possibly could by avoiding health threats (e.g. infections) (Table 2, quote 1C). They searched for the best possible treatments and support, tried to control as best they could their child’s symptoms and disease and preferably took care of the child themselves. By doing so, parents tried to control their anxiety.

(2) *Confrontations with loss and related grief*

Apart from the threat of definitely losing their child, parents emphasised that they continuously had to deal with feelings of loss and grief related to their child’s deterioration (Table 2, quote 2A). They had to constantly adapt their own life to fulfil their parental role of caregiving. Every moment of loss, as experienced by the individual parent, meant that they had to go through a mourning cycle, again and again. For instance, situations when their child was no longer able to attend school, to eat independently, or to communicate, were experienced as losses and triggered grief. Parents felt also confronted with loss and grief because of the continuous necessity of adjusting one’s own goals, for example by letting go of the dream of having a carefree family life (Table 2, quote 2B,C). Moreover, many parents experienced limited opportunities to continue and develop their own life, such as limitations in their career options, lack in time for hobbies and loss of contact with relatives and/or friends (Table 2, quote 2D). Therefore, many parents felt that their life, or at least part of it, was ‘on hold’.

(3) *Ambiguity towards uncertainty*

Many parents experienced their child’s disease trajectory as unpredictable, also in the face of approaching death. The lack of clear information from HCP’s on expected or possible child development, functional performance or duration of life contributed greatly to feelings of uncertainty and lack of control (Table 2, quote 3A). The only certainty parents experienced was the inevitable premature death of their child, without exactly knowing when death would occur (Table 2, quote 3B).

Parents reported ambiguous needs regarding uncertainty (Table 2, quote 3C). On the one hand, they preferred concrete information on the expected disease trajectory, even concerning their child’s life expectancy. This information could support future perspectives guiding them in the organisation of their lives, including family life and emotions. On the other hand, parents tended to avoid concrete information and appraised the uncertainty positively because it enabled them to preserve hope and enjoy the here-and-now in daily life with their child. Both coping styles were observed in the individual parent’s stories, reflecting their ambiguity. It varied among parents and situations according to whether the need for concrete information, or the wish to cherish uncertainty, predominated.

(4) *Preservation of a meaningful parent-child relationship*

Parents experienced their unique significance to their child by taking responsibility for its well-being and by ‘being there’ for their child (Table 2, quote 4A). The feeling of being of worth to their child’s well-being helped them to take care of the child despite the complexity and burden of the circumstances. Parents reflected on what their child meant to them and reported that they loved behaviour showing their child’s character. As such, recognition of their child’s personality strengthened their meaningful parent-child relationship, in particular for the NMD group. Experiencing this meaningfulness was of major importance to parents because it showed the essence of being a ‘good parent’ of a child with a LLD/LTD (Table 2, quote 4B). For some parents, the meaningful parent-child relationship became threatened when the interaction with their child altered or when their child’s personality became less apparent due to disease progression. As such, they experienced that caring for their child became more demanding and harder to maintain.

(5) *Tension regarding end-of-life decisions*

For almost all parents, it was of major importance to preserve their child’s life. However, the deterioration of their child’s physical condition raised questions for them about whether their child was sufficiently comfortable (Table 2, quote 5A). Parents knew that, ultimately, they had to let go of their child, but at the same time, they wanted to keep their beloved child with them. Therefore, making decisions regarding the end of their child’s life, in particular decisions that allowed this phase to proceed, came with great tension (Table 2, quote 5B). Despite these tensions, a few parents, nevertheless, urged HCPs to discuss end-of-life decisions with them, from both a perspective of holding on to life and of letting it go. Some other parents, who anticipated their child’s end-of-life phase or who were currently in it, did not want to be forced to make a difficult end-of-life decision themselves and preferred to see their child as having a more active role. For example, after a treatment was started they expected their child to ‘demonstrate’ his or her ‘choice’ by either recovering or not. Parents also expressed the hope that their child would ‘choose’ a moment to suddenly pass away, so that they would not have to make that decision (Table 2, quote 5C).

(6) *Engagement with professionals*

Parents had mixed experiences with their relationships with healthcare professionals and with the healthcare system in general. Positive experiences involved HCPs who had experience and expertise in PPC and who tried to provide care which matched their child’s personality. Parents also viewed HCPs positively who were able to align their care with their child’s and own needs (Table 2, quote 6A). Disappointing experiences involved a lack of coordination and of continuity of care, feelings of abandonment, particularly when there was no treatment available, and being sent home without care arrangements after the diagnosis of an incurable disease (Table 2, quote 6B). Adding to the burden of care was the loss of their privacy when homecare was involved, and the necessity to learn both the speech and body language of the doctors in order to understand the real messages hidden in their words (Table 2, quote 6C).

### Coping strategies

Four main coping strategies could be determined: (1) suppressing emotions; (2) seeking support; (3) taking control; and (4) adapting and accepting.

1. *Suppressing emotions*

Parents noted that facing the loss of their child triggered intense emotions. They tried, however, to suppress these feelings by avoiding thinking about their child’s premature death, simply in order to maintain an, as normal as possible, daily life and to be able to do what needed to be done (Table 3, quote 1A). In succeeding, they were able to fulfil their parenting and caring roles, create a positive atmosphere and enjoy daily life (Table 3, quote 1B). In terms of a metaphor, one might think of this as an imaginary screen allowing parents to make the most of their daily lives by keeping the difficulties of an approaching death out of sight, while not denying its existence.

Even so, the threat of losing their child still regularly invaded parents’ thoughts, especially when the loss of their child was becoming more tangible. This happened, for example, during intermittent crises in the course of the disease and during the end-of-life-process. Parents reported the use of additional cognitive and behavioural coping strategies, such as focussing on the ‘here-and-now’, maintaining daily routines, preserving hope and thinking positively by focussing on what is still possible rather than on problems (Table 3, quote 1C). These strategies strengthened them in keeping the thoughts of their child’s death at bay. Even during the end-of-life phase, parents seemed able to suppress thoughts of life without their child, enabling them to take care of, and be there for, their child, while postponing their own emotional needs and anticipatory grief.

(2) *Seeking support*

Parents did not always experience support as being in tune with their needs and preferences throughout the palliative trajectory. This was in spite of the availability of a wide range of aspects from which support could be derived. Parents felt supported when they were acknowledged in their feelings or when they could share their story about what they had gone through. Also, others who helped them in childcare, but without taking over their unique parenting role, were considered supportive. Most parents mentioned their spouse as their main source of support and empowerment.

From the start of their child’s disease process, most parents showed a tendency to withdraw from social life and focus on caregiving and maintaining a family balance. Parents often allowed only a few significant others into their inner circle, such as close friends, relatives and/or peers who were supportive and trustworthy in their care (Table 3, quote 2A). In addition to social support, many parents felt strengthened by their religion or faith, or by finding distraction in work, studying, hobbies, sports or writing a blog (Table 3, quote 2B,C).

(3) *Taking control*

During the disease process, parents gradually became familiar with the new world surrounding their ill child. They gained insight into their priorities and preferences concerning their child and family. Parents increasingly realised that they were the continuous factor in their child’s care, and being so, they felt they best knew their child’s needs. They also learned that care wasn’t provided automatically and that HCPs did not always give priority to their child and family.

Parents stated that these experiences urged them to take control by arranging a care situation that fitted their child and family situation (Table 3, quote 3). For instance, building a team of healthcare professionals surrounding their child whom you would prefer and which delivered continuity and expertise. Parents actively guided the HCPs to provide personalised care for their child that did justice to his or her personality. Although taking control reduced emotional distress, parents often experienced it as a difficult and exhausting task.

(4) *Adapting and accepting*

Many parents found ways to accept the changes in life that came along with being the parent of a child with a LLD/LTD (Table 3, quote 4A). This acceptance helped parents to create a state that could be indicated as the new normal. It consisted of changing their priorities and adapting life plans for all family members involved in order to fulfil the caregiving tasks needed and to carry on with life (Table 3, quote 4B). This process of adaptation towards the new normal was experienced as an emotional effort in which most parents felt they had made sacrifices. Observing the strength and resilience of their child was helpful in this process as was the positive reinforcement of their parental behaviour by their child, family members or HCPs. A few parents mentioned that the rewarding aspects of their caregiving role outweighed the burden of their being forced to adapt their lives which lead to feeling privileged in comparison to other parents (Table 3, quote 4C). Losing highly valued goals or future dreams, on the other hand, could compromise this adaptation and acceptance.

Throughout the palliative trajectory, their child’s development, changes in condition together with changes in healthcare or in financial circumstances, imposed new challenges to parental adaptation (Table 3, quote 4D,4E). Therefore, parental acceptance was not a permanent state of being. It required continuously adapting to new circumstances.

## Discussion

Every day parents of children in need of PPC suffered from both the anxiety of losing their child and from being confronted regularly with loss and related grief. They experienced an ambiguity concerning their uncertain circumstances. While they worked towards and cherished a meaningful relationship with their child, they also felt tension regarding end-of-life decisions and in their engagement with HCPs. Four main coping strategies were identified closely related to the parents’ experiences: suppressing emotions, seeking support, taking control, and continuously adapting their life to the ongoing changes.

This study shows that all parents felt the anxiety of losing their child every day, even when their child is doing relatively well and when the end of their child’s life is still far away. In line with previous studies, parents feel confronted with past, current or anticipated losses related to their child’s and their own life [4, 15, 34]. Our study adds that parenting a child with a LLD/LTD implies a continuous management of feelings of anxiety and loss. Without ignoring the fact that their child will die sooner or later, parents suppress concrete thoughts about the imminent loss of their child in order to provide dedicated childcare, to arrange family life and to perform the task of parenting. Suppressing their emotions by adopting avoidant coping strategies also helps them to maintain a degree of optimism and to carry on in daily life [38]. In the literature, this is also referred to as *healthy denial* [7, 23]. It is hypothesised that this mechanism of healthy denial could at times make parents hesitate before discussing their child’s future with HCPs. Cultivating prognostic uncertainty allows them to postpone, to some extent, looking forward and thus facing up to the child’s deteriorating health.

For HCPs, it is considered important to align their communication on current and future issues regarding the child and family with the needs of the parents. But also, in addition to this, to schedule these types of conversations in consultation with the parents. The reason is that tension might exist between proactively discussing and organising care for the child and family on the one hand and, on the other hand, respecting the parents’ avoidant strategies. Scheduling decisive conversations in advance offers parents the opportunity to prepare for difficult conversations in order not to disrupt them more than necessary. By doing so, HCPs support effective ways for parents to cope with the future loss of their child, which is indicated as a necessity to be titrated in bereavement literature [41]. Furthermore, entering the lives of families with a child with a LLD/LTD, and providing adequate support, requires specific sensitivity and skills from HCPs. Correctly interpreting parental values, sensing perceived losses and respecting individual coping strategies seem necessary to establish an adequate working relationship. It is important to obtain consensus with parents on when to discuss future care, while realising that couples might hold different views on the timing [44]. Finding a careful balance between respecting individual strategies and a process of communication on future care, known as advance care planning, will help to improve the quality of PPC [8, 24, 36, 51].

For most parents experiencing a meaningful relationship with their child and aligning care to his or her needs is of major importance. In line with others, experiencing a sense of meaning increases parents’ resilience and reinforces their ability to care throughout the palliative trajectory of their child [10, 50]. As such, it might be helpful if HCPs advise parents, from the start of the disease process, how best to preserve and support the manifestations of a meaningful relationship with their child.

Although many parents receive support from their spouse and/or from a few significant others, the social support system of many parents becomes smaller. The feeling that other relatives and friends do not understand their situation and the burden seems to drive people apart [21]. Still, some parents are able to enlarge their social life again. Connecting with peers is experienced as especially valuable by parents, as shown too by Nicholas et al. [29] Social support has been identified as one of the most common and effective strategies for coping with stress and for maintaining psychological and physical well-being [6, 19, 30, 37, 39]. HCPs should, therefore, stimulate parents to seek social support and assist them to have contact with peers.

Parents act as protectors and advocates for their child as they are convinced that their ill child depends on them and that they know their child and his or her situation best. They do so by gradually taking the initiative in arranging the care in such a way as to ensure the child’s needs are met. Other studies have shown this too [2, 4, 12, 53]. Though taking control requires effort, it also reduces parents’ distress because they know the care will be arranged in the way they prefer. Additionally, both receiving social support and taking the initiative in directing care appear to be a new skill for parents. Therefore, HCPs can help parents to learn these new skills by identifying and supporting their coping strategies.

This study had some strengths and limitations. Just as in most palliative care studies, the HCPs acted as gatekeepers to the study by making a judgement that some parents, though eligible, should not be recruited because they considered them too vulnerable, or carrying too heavy a burden, for participation in research [16]. Hence, the needs of these families are under-represented in this sample. The sample consisted of mainly native Dutch-speaking parents. It was not able to embrace different cultural backgrounds. Future research should investigate the experiences of parents from different cultural backgrounds. In addition, while the Netherlands generally enjoys relatively high levels of education, well-educated Dutch-speaking parents were still over-represented. We found that all parents struggle with similar experiences in daily life and that the main coping strategies are also quite similar. However, highly educated parents might have different skills and resources in coping with their situation and organising the best care for their child. As such the solutions chosen could differ in practice. Nonetheless, we included the perspectives of both mothers and fathers, and our sample showed a wide variation in diagnoses and stages of the palliative trajectory. This ranged from currently caring for a child, to recently having lost a child, which improved the validity of the study’s conclusions.

## Conclusion

This study reports in detail about the most prominent parental experiences when caring for a child with a LLD/LTD, both MD and NMD, at home. It also investigates parents coping strategies in adjusting to the situation. Parenting and caring for a child with a LLD/LTD require continuous management of anxiety and loss. At the same time, parents work towards a new normality and gradually take control to arrange the best care for their child and family. Some parents manage this process well, while for other parents, this process is a major burden and they need adequate support from HCPs. In order to provide support and guidance geared towards specific families from the start of the disease trajectory, HCPs need to understand parents’ anxiety, grief, relationship with their child and coping strategies.

## Electronic supplementary material

ESM 1 (DOCX 18 kb)

## Abbreviations

HCP: healthcare professional
LLD: life-limiting disease
LTD: life-threatening disease
MD: malignant disease
NMD: non-malignant disease
PPC: paediatric palliative care
PPCT: paediatric palliative care team
